# Do Connexin Mutants Cause Cataracts by Perturbing Glutathione Levels and Redox Metabolism in the Lens?

**DOI:** 10.3390/biom10101418

**Published:** 2020-10-07

**Authors:** Oscar Jara, Peter J. Minogue, Viviana M. Berthoud, Eric C. Beyer

**Affiliations:** Department of Pediatrics, University of Chicago, Chicago, IL 60637, USA; ojaraleiva@peds.bsd.uchicago.edu (O.J.); pminogue@peds.bsd.uchicago.edu (P.J.M.); vberthou@peds.bsd.uchicago.edu (V.M.B.)

**Keywords:** connexin, cataracts, oxidative stress, GSH metabolism

## Abstract

Cataracts of many different etiologies are associated with oxidation of lens components. The lens is protected by maintenance of a pool of reduced glutathione (GSH) and other antioxidants. Because gap junction channels made of the lens connexins, Cx46 and Cx50, are permeable to GSH, we tested whether mice expressing two different mutants, Cx46fs380 and Cx50D47A, cause cataracts by impairing lens glutathione metabolism and facilitating oxidative damage. Levels of GSH were not reduced in homogenates of whole mutant lenses. Oxidized glutathione (GSSG) and the GSSG/GSH ratio were increased in whole lenses of Cx50D47A, but not Cx46fs380 mice. The GSSG/GSH ratio was increased in the lens nucleus (but not cortex) of Cx46fs380 mice at 4.5 months of age, but it was not altered in younger animals. Carbonylated proteins were increased in Cx50D47A, but not Cx46fs380 lenses. Thus, both mouse lines have oxidizing lens environments, but oxidative modification is greater in Cx50D47A than in Cx46fs380 mice. The results suggest that GSH permeation through lens connexin channels is not a critical early event in cataract formation in these mice. Moreover, because oxidative damage was only detected in animals with significant cataracts, it cannot be an early event in their cataractogenesis.

## 1. Introduction

Gap junction-mediated intercellular communication has important roles in the lens. The lens is an avascular organ. The bulk of it is formed by fiber cells that lose their organelles during the differentiation of surface epithelial cells at the equator. The survival of lens fiber cells depends on an internal circulation, which is responsible for the flow of water, ions, and small molecules through the lens. In the lens internal circulation, water, ions and small solutes enter the lens through the anterior and posterior poles and exit at the equator. Gap junction channels provide the pathway for the outflow of water, ions, and solutes across cell boundaries (reviewed in [1]). 

Lens fiber cell gap junctions are formed by two different subunit proteins, connexin46 (Cx46) and connexin50 (Cx50). Mutations of the genes encoding Cx46 and Cx50 cause cataracts in humans and mice [2]. Analysis of cataract-linked connexin mutants may yield substantial insight into the critical functions of wild-type connexins. 

A cataract is a cloudiness in the lens that causes a decrease in vision by distorting, scattering, or blocking light transmission; thereby preventing proper focusing of an image on the retina. Although lens opacities can result from different etiologies, a central feature is damage to lens cells and their components. In many studies of cataractous lenses, this damage includes oxidation of lens proteins and lipids [3,4,5]. The preservation of transparency throughout life depends on the presence of endogenous anti-oxidants in the lens, especially reduced glutathione (GSH) [6,7,8,9]. GSH levels are maintained through the activity of key enzymes, but only superficial nucleated cells have the synthetic capacity to produce reduced glutathione. Because gap junction channels allow the direct intercellular passage of ions and other small solutes, it has been hypothesized that they may be critical for the delivery of GSH to internal lens cells. Slavi et al. [10] demonstrated that GSH can permeate through both Cx46 and Cx50 channels, and they suggested that Cx46 containing gap junction channels are required for the delivery of GSH to central lens fiber cells. Other studies have also suggested links between lens connexin function and glutathione metabolism [11].

We sought to test whether connexin mutants cause cataracts by disturbing levels and metabolism of glutathione, leading to oxidative damage. We utilized mouse lines that model human congenital cataracts due to expression of a Cx50 point mutation (Cx50D47A) or a Cx46 frameshift mutation (Cx4fs380). The lenses of both lines develop cataracts. In both mouse lines, lenses have severely reduced levels of Cx46 and Cx50, drastically impaired lens cell–cell communication, increased hydrostatic pressure, and accumulation of Ca^2+^ [12,13,14,15,16]. However, there are also several differences between these two models (summarized in Table 1). Differentiation and growth are not very different between lenses of Cx46fs380 and wild-type mice, but these processes are impaired in Cx50D47A mutant lenses. The timing of cataract development is different between lines: overt nuclear opacities are readily detectable in both homozygous and heterozygous Cx50D47A mice at 1 month of age, but opacities (anterior nuclear) are not observed until ~2 months (homozygotes) and ≥4 months (heterozygotes) in Cx46fs380 mice. At any given age, the cataracts are more severe in Cx50D47A mice than in Cx46fs380 mice. Because of these differences, we studied glutathione levels and other aspects of redox metabolism at different ages in these two mutant lines to determine if and when abnormalities are present and whether they change with cataract development or progression.

## 2. Materials and Methods

### 2.1. Animals

Cx50D47A (also known as No2 or ENU-326) mutant mice [17] were maintained in the C3H strain as previously reported [13]. Cx46fs380 animals were generated and maintained as described by Berthoud et al. [12]. All animal procedures were approved by the University of Chicago Animal Care and Use Committee and followed its guidelines.

### 2.2. Measurement of Reduced and Oxidized Glutathione (GSH and GSSG)

Levels of total GSH and GSSG were determined using the GSH/GSSG-Glo^TM^ Assay (V6611, Promega, Madison, WI, USA) based on the GSH-dependent conversion of a GSH probe, luciferin-NT, to luciferin by glutathione-S-transferase coupled to a firefly luciferase reaction, following the manufacturer’s instructions with minor modifications. Whole lenses or the lens cortex and nucleus (pooled from both eyes) were dissected from individual 1- and 2.5-month-old wild-type, 1- and 2.5-month-old Cx50D47A, or 2.5-month-old Cx46fs380 mice. For 4.5-month-old mice, a single whole lens or lens cortex or nucleus was used. The dissected tissues were homogenized in PBS (pH = 7.4) containing 2 mM EDTA. After a 5-min cycle of 30 s ON and 30 s OFF at 4 °C in a bath sonicator (Bioruptor, UCD-200, Diagenode, Denville, NJ), proteins and other large molecular weight material were removed from the samples by centrifugation for 20 min at 16,000× *g* at 4 °C using a filter unit with a 3 kDa cut-off (Amicon ultra-0.5 mL, UFC500324, EMD Millipore, Billerica, MA, USA). The flowthrough was collected and used to perform the assay using a TD-20/20, 2020-998 luminometer (Turner Biosystems, Sunnyvale, CA, USA). The protein concentration of the initial homogenates was determined using the BioRad Protein Assay (BioRad, Hercules, CA, USA) based on the method of Bradford [18]. The values are reported as pmol of GSH per µg of protein in the lens homogenate. 

### 2.3. Antibodies

Mouse monoclonal anti-glutathione synthetase (sc-365863, lot no. D1913) and anti-glutathione reductase (sc-133245, lot no. C0119) antibodies were obtained from Santa Cruz Biotechnology, Inc. (Dallas, TX, USA). Horseradish peroxidase-conjugated AffiniPure F(ab’)_2_ fragment goat anti-mouse IgG, F(ab’)_2_ fragment-specific (115-036-072; lot no. 139645) antibodies were obtained from Jackson ImmunoResearch (West Grove, PA, USA). 

### 2.4. Polyacrylamide Gel Electrophoresis, Immunoblotting, and Silver Staining

After dissection, lens samples obtained from Cx50D47A and Cx46fs380 mice were homogenized in 1X PBS (pH = 7.4) containing 4 mM EDTA, 20 mM NaF, and cOmplete Mini EDTA-free Protease Inhibitor Cocktail (Roche Applied Science, Indianapolis, IN, USA) followed by sonication. Protein concentrations were determined as described above or using the QuantiPro™ BCA Assay Kit (QPBCA; Millipore Sigma, Burlington, MA, USA) for the silver staining experiments described below. Aliquots with equal amounts of total proteins were resolved by sodium dodecyl sulfate-containing polyacrylamide gel electrophoresis (SDS-PAGE). 

For immunoblotting, proteins were resolved on 10% polyacrylamide gels, then electrotransferred to Immobilon P (Millipore Sigma) and stained with Ponceau S to verify equal loading and transfer of proteins as previously described [19]. Subsequently, membranes were rinsed and subjected to immunoblotting as previously described [20]. Briefly, membranes were incubated with a 1:250 dilution of mouse monoclonal anti-glutathione synthetase or a 1:500 dilution of mouse monoclonal anti-glutathione reductase antibodies overnight at 4 °C. Then, membranes were rinsed in Tris-buffered saline pH = 7.4 (TBS), incubated with a 1:1000 dilution of horseradish peroxidase-conjugated goat anti-mouse antibodies for 1.5 h followed by TBS rinses. Binding of secondary antibodies was detected by enhanced chemiluminescence using the GE Healthcare Amersham^TM^ ECL^TM^ Western Blotting Detection Reagents (45000885, Thermo Fisher Scientific, Waltham, MA, USA). Densitometric analysis of the bands was performed using Adobe Photoshop CS3 (Adobe Systems Inc., San Jose, CA, USA). The results are reported in arbitrary units.

For silver staining detection of all proteins in a sample, 30 μg of lens homogenate proteins were resolved on 8% polyacrylamide gels under reducing (β-mercaptoethanol added to sample) and non-reducing conditions (aliquot without addition of β-mercaptoethanol). After electrophoresis, the gels were rinsed and fixed in a 30% ethanol:10% acetic acid solution. Fixed gels were stained for 30 min at room temperature using the Pierce Silver Stain Kit (24612, Thermo Fisher Scientific) and then developed for 2–3 min until the bands appeared. The reaction was stopped using a 5% acetic acid solution. The high molecular weight material encompassing the gel region from ~180 kDa to the top of the resolving gel was quantified by densitometry to determine the mean grey value. 

### 2.5. Detection and Quantification of Carbonylated Proteins

Protein carbonylation was assessed using the Protein Carbonyl Assay Kit (ab178020, Abcam, Cambridge, MA, USA). Briefly, homogenates from freshly dissected lenses from 1- and 2.5-month-old Cx50D47A mice and 2.5- and 4.5-month-old Cx46fs380 mice were prepared, and the protein concentrations were determined as described in Section 2.2. Equal amounts of proteins from each lens homogenate were incubated with 2,4-dinitrophenylhydrazine to modify the carbonyl groups in the proteins with a 2,4-dinitrophenyl (DNP) group. The derivatized proteins were resolved by SDS-PAGE using 15% polyacrylamide gels and electrotransferred onto Immobilon P. The membranes were incubated with a 1:5000 dilution of the rabbit anti-DNP antibodies followed by a 1:5000 dilution of the horseradish peroxidase-conjugated goat anti-rabbit IgG antibodies provided in the kit. Densitometric analyses were performed using Adobe Photoshop CS3 by drawing a rectangular box encompassing the entire lane.

### 2.6. Statistical Analysis 

Raw data obtained from lenses of heterozygous and homozygous mutant mice were compared with the raw data obtained from wild-type mice to assess statistical significance using Student’s *t*-test. Data are presented as the mean + standard error of the mean. Graphs were prepared using GraphPad Prism 8 (GraphPad Software, San Diego, CA, USA). A *p* value ≤ 0.05 was considered significant. The number of sets of mice containing all genotypes (i.e., statistical “n”) was at least three for each type of data presented. Since many of the sets were comprised of littermates, gender matching within each set was not always possible.

## 3. Results

### 3.1. GSH Levels are Not Reduced in Connexin Mutant Lenses

**Figure 1:**
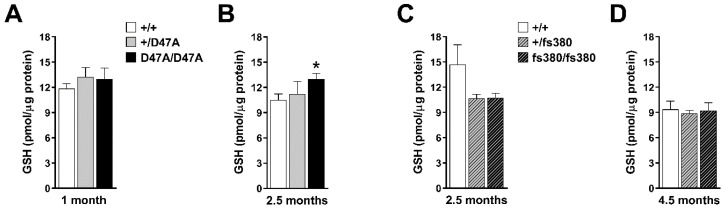
Levels of reduced glutathione (GSH) in Cx50D47A and Cx46fs380 lenses. (**A**,**B**) Graphs show the levels of GSH in 1- (**A**) and 2.5- (**B**) month-old wild-type (+/+), and heterozygous (+/D47A) and homozygous (D47A/D47A) Cx50D47A mice. (**C**,**D**) Graphs show the levels of GSH in 2.5- (**C**) and 4.5- (**D**) month-old wild-type (+/+) and heterozygous (+/fs380) and homozygous (fs380/fs380) Cx46fs380 mice. Data are presented as the mean (bar) + standard error of the mean (n = 3 for 1-month-old and n = 4 for 2.5-month-old Cx50D47A lenses; n = 3 for 2.5-month-old and n = 4 for 4.5-month-old Cx46fs380 lenses). The asterisk denotes a significant difference compared with wild-type littermates (*p* < 0.05). GSH levels in 2.5-month-old fs380 lenses were not significantly different from wild-type (*p* = 0.19 for wild-type vs. heterozygotes, and *p* = 0.25 for wild-type vs. homozygotes).

We determined GSH levels in whole lens homogenates from 1- and 2.5-month-old Cx50D47A mice and from 2.5- and 4.5-month-old Cx46fs380 mice. We calculated cellular concentrations based on protein amounts (pmol GSH/µg protein); this allowed direct comparison of values between wild type and mutant lenses, independent of differences in lens sizes. Surprisingly, we did not find reductions in GSH concentrations in any of the mutant lenses (Figure 1A–D). Indeed, the only significant difference was in homogenates from homozygous Cx50D47A mice at 2.5 months of age in which levels of GSH were increased by 26% as compared to wild-type lenses of the same age (Figure 1B). 

### 3.2. Enzymes for GSH Production Are Increased in Cx50D47A and Cx46fs380 Lenses

The increase in levels of GSH in 2.5-month-old homozygous Cx50D47A lenses raised the possibility that the levels of the enzymes responsible for its synthesis and the maintenance of its reduced state were altered. To test this, we evaluated the levels of glutathione synthetase and glutathione reductase in Cx50D47A lenses at 1 and 2.5 months of age and in Cx46fs380 lenses at 2.5 and 4.5 months of age. 

**Figure 2:**
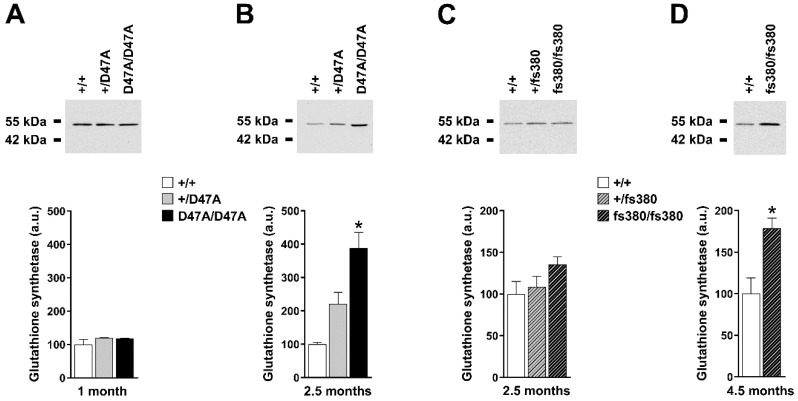
Levels of glutathione synthetase in Cx50D47A and Cx46fs380 lenses. (**A**,**B**) Top panels. Immunoblots of glutathione synthetase in whole lens homogenates from 1- (**A**) and 2.5- (**B**) month-old mice that were wild type for Cx50 (+/+) or heterozygous (+/D47A) or homozygous (D47A/D47A) for Cx50D47A. The migration positions of the molecular mass markers are indicated on the left. Bottom panels. Graphs show the densitometric quantification of the glutathione synthetase immunoreactive band obtained in 3 independent experiments. Data are presented as the mean (bar) + standard error of the mean. (**C**,**D**) Top panels. Immunoblots of glutathione synthetase in whole lens homogenates from 2.5- (**C**) and 4.5- (**D**) month-old mice that were wild type for Cx46 (+/+) or heterozygous (+/fs380) or homozygous (fs380/fs380) for Cx46fs380. The migration positions of the molecular mass markers are indicated on the left. Bottom panels. Graphs show the densitometric quantification of the glutathione synthetase immunoreactive band obtained in three independent experiments. Data are presented as the mean (bar) + standard error of the mean. Asterisk denote a significant difference compared with wild-type lenses (*p* < 0.05). Levels of glutathione synthetase in 2.5-month-old heterozygous Cx50D47A lenses were not significantly different than wild-type (*p* = 0.07).

**Figure 3:**
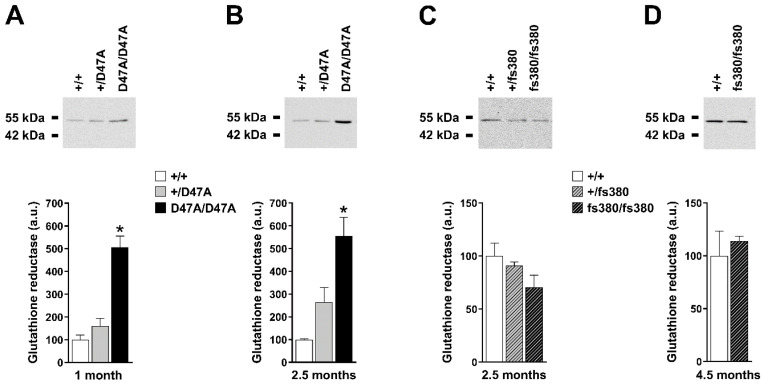
Levels of glutathione reductase in Cx50D47A and Cx46fs380 lenses. (**A**,**B**) Top panels. Immunoblots of glutathione reductase in whole lens homogenates from 1- (**A**) and 2.5- (**B**) month-old mice that were wild type for Cx50 (+/+) or heterozygous (+/D47A) or homozygous (D47A/D47A) for Cx50D47A. The migration positions of the molecular mass markers are indicated on the left. Bottom panels. Graphs show the densitometric quantification of the glutathione reductase immunoreactive band obtained in three independent experiments. Data are presented as the mean (bar) + standard error of the mean. (**C**,**D**) Top panels. Immunoblots of glutathione reductase in whole lens homogenates from 2.5- (**C**) and 4.5- (**D**) month-old mice that were wild type for Cx46 (+/+) or heterozygous (+/fs380) or homozygous (fs380/fs380) for Cx46fs380. The migration positions of the molecular mass markers are indicated on the left. Bottom panels. Graphs show the densitometric quantification of the glutathione reductase immunoreactive band obtained in three independent experiments. Data are presented as the mean (bar) + standard error of the mean. Asterisk denote a significant difference compared with wild-type lenses (*p* < 0.05). Note that at 2.5 months of age, levels of glutathione reductase in heterozygous Cx50D47A lenses (*p* = 0.12), heterozygous Cx46fs380 lenses (*p* = 0.54) and homozygous Cx46fs380 lenses (*p* = 0.15) were not different from those in wild-type lenses.

Levels of glutathione synthetase did not differ significantly among genotypes in 1-month-old Cx50D47A mice, but they were increased almost 4-fold in lenses of 2.5-month-old homozygous Cx50D47A mice (Figure 2A,B). Modest increases of glutathione synthetase at 2.5 months in heterozygous Cx50D47A lenses did not reach statistical significance. We found no differences in levels of glutathione synthetase in either heterozygous or homozygous Cx46fs380 lenses at 2.5 months; however, they were increased 1.8-fold in 4.5-month-old homozygous Cx46fs380 lenses (Figure 2C,D). Levels of glutathione reductase were increased about 5-fold in homozygous Cx50D47A mice at both 1 and 2.5 months of age (Figure 3A,B). Levels of glutathione reductase were not significantly different between wild-type and heterozygous Cx50D47A lenses at either age. In Cx46fs380 lenses, levels of glutathione reductase were not significantly different among genotypes at 2.5 or at 4.5 months of age (Figure 3C,D).

### 3.3. Oxidation of Glutathione is Greater in Cx50D47A than in Cx46fs380 Lenses

**Figure 4:**
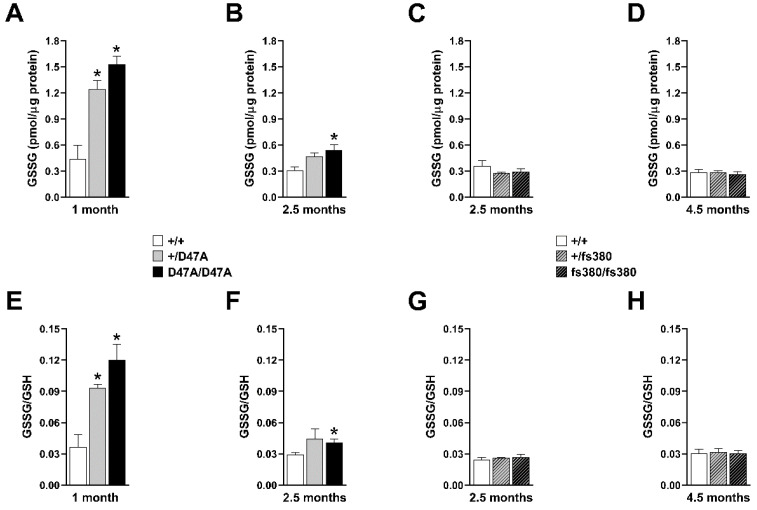
GSSG levels and GSSG/GSH ratio in whole lenses from Cx50D47A and Cx46fs380 mice. (**A**,**B**,**E**,**F**) Graphs show the levels of GSSG (**A**,**B**) and the GSSG/GSH ratio (**E**,**F**) in lenses from 1- (**A**,**E**) and 2.5- (**B**,**F**) month-old mice that were wild type for Cx50 (+/+), and heterozygous (+/D47A) and homozygous (D47A/D47A) for Cx50D47A. (**C**,**D**,**G**,**H**) Graphs show the levels of GSSG (**C**,**D**) and GSSG/GSH ratio (**G**,**H**) in lenses from 2.5- (**C**,**G**) and 4.5- (**D**,**H**) month-old wild-type (+/+) and heterozygous (+/fs380) and homozygous (fs380/fs380) Cx46fs380 mice. Data are presented as the mean (bar) + standard error of the mean (n = 3 for 1-month-old and n = 4 for 2.5-month-old Cx50D47A mice; n = 3 for 2.5-month-old Cx46fs380 mice and n = 4 for 4.5-month-old Cx46fs380 mice). Asterisk denote a significant difference compared with wild type (*p* < 0.05). GSSG levels and the GSSG/GSH ratio were not significantly different between wild-type and 2.5-month-old heterozygous Cx50D47A lenses (*p* = 0.09 and 0.24, respectively).

To assess the oxidation of glutathione in Cx50D47A and Cx46fs380 lenses, we determined the levels of oxidized glutathione (GSSG) and the GSSG/GSH ratio in whole lenses and compared these values with those obtained in wild-type mice. Levels of GSSG in both heterozygous and homozygous Cx50D47A lenses were significantly increased as compared with wild-type lenses of 1-month old mice (Figure 4A). At 2.5 months, levels of GSSG were significantly increased in homozygous Cx50D47A lenses as compared with wild-type, but the apparent increase in heterozygotes did not reach statistical significance (Figure 4B). The GSSG/GSH ratio was significantly increased at 1 month in heterozygous Cx50D47A lenses and at both 1 and 2.5 months in homozygous Cx50D47A lenses (Figure 4E,F) as compared with wild type. At 2.5 months, the GSSG/GSH ratio was not significantly different between heterozygotes and wild-type lenses (Figure 4F). In contrast, neither the GSSG levels nor the GSSG/GSH ratio in whole lens homogenates of Cx46fs380 mice differed from wild-type values at either 2.5 (Figure 4C,G) or 4.5 months of age (Figure 4D,H). 

### 3.4. GSH levels are Decreased and Its Oxidation Is Increased in the Nucleus of Older Cx46fs380 Lenses

**Figure 5:**
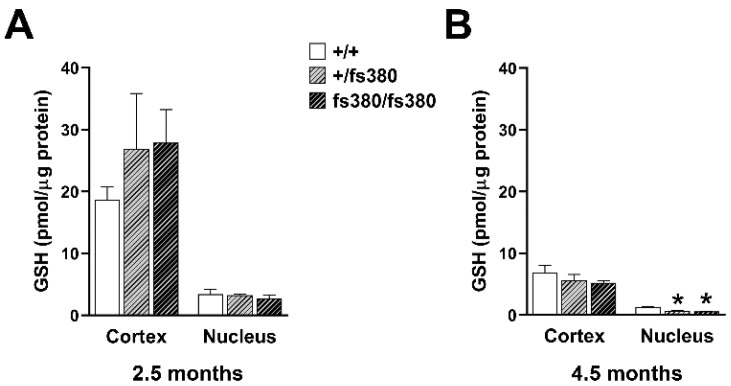
Levels of GSH in the cortex and nucleus of Cx46fs380 lenses. (**A**,**B**) Graphs show the levels of GSH in the lens cortex and nucleus from wild-type (+/+) and heterozygous (+/fs380) and homozygous (fs380/fs380) Cx46fs380 mice at 2.5 (**A**) and 4.5 (**B**) months of age. Data are presented as the mean (bar) + standard error of the mean (n = 3 for 2.5-month-old lenses and n = 4 for 4.5-month-old lenses). Asterisk denote a significant difference compared with wild-type littermates (*p* < 0.05). GSH levels in the cortex of 4.5-month-old heterozygous and homozygous Cx46fs380 lenses were not different from wild-type (*p* = 0.46 for wild-type vs. heterozygotes, and *p* = 0.25 for wild-type vs. homozygotes).

Because we did not observe differences in the levels of reduced or oxidized glutathione in whole lenses of Cx46fs380 mice, we considered that there might be regional differences, especially if expression of the mutant impaired delivery of GSH to the central region of the lens. To test this hypothesis, we determined GSH levels in homogenates from lens cortex and nucleus of Cx46fs380 mice. Similar to previous studies [10,21,22,23], we found that GSH levels were much higher in the cortex than in the nucleus. Regardless of genotype, GSH concentrations were 5–10-fold higher in cortical homogenates than in nuclear homogenates (Figure 5). At 2.5 months, GSH levels did not differ significantly between wild-type and Cx46fs380 (heterozygous or homozygous) lenses in either cortex or nucleus (Figure 5A). At 4.5 months, GSH levels in the cortex of heterozygous and homozygous Cx46fs380 lenses were similar to those in wild-type lenses; however, they were significantly reduced in the lens nucleus of both heterozygous and homozygous Cx46fs380 mice (Figure 5B).

**Figure 6:**
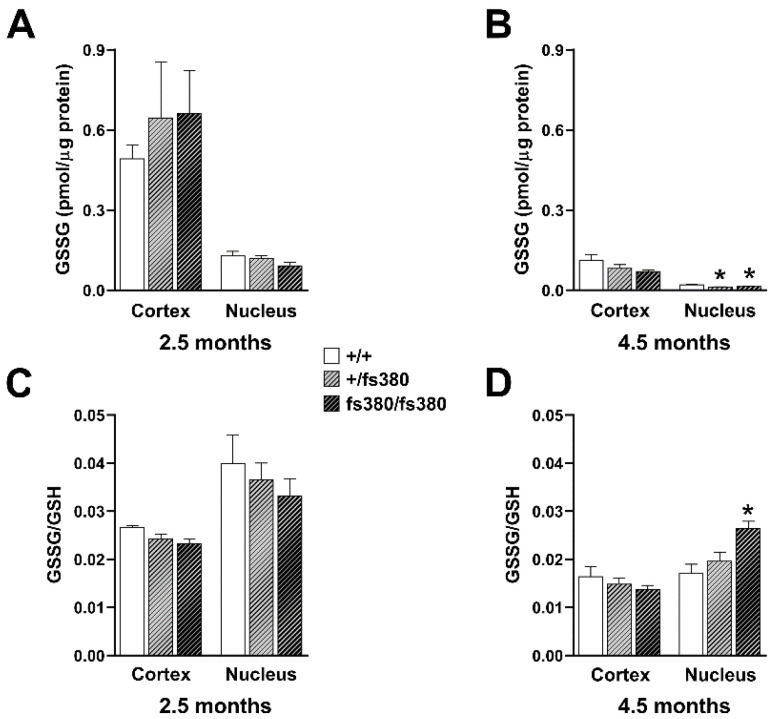
GSSG levels and GSSG/GSH ratio in the cortex and nucleus of Cx46fs380 lenses. (**A**,**B**) Graphs show the GSSG levels in the lens cortex and nucleus from wild-type (+/+) and heterozygous (+/fs380) and homozygous (fs380/fs380) Cx46fs380 mice at 2.5 (**A**) and 4.5 (**B**) months of age. (**C**,**D**) Graphs show the GSSG/GSH ratio in the lens cortex and nucleus from wild-type (+/+) and heterozygous (+/fs380) and homozygous (fs380/fs380) Cx46fs380 mice at 2.5 (**C**) and 4.5 (**D**) months of age. Data are presented as the mean (bar) + standard error of the mean (n = 3 for 2.5-month-old Cx46fs380 mice and n = 4 for 4.5-month-old Cx46fs380 mice). Asterisk denote a significant difference compared with wild type (*p* < 0.05). GSSG levels in homozygous Cx46fs380 lenses were not significantly different from wild-type in the cortex at 2.5 months and 4.5 months of age (*p* = 0.54 and *p* = 0.09, respectively), nor did they differ in the nucleus at 2.5 months of age (*p* = 0.14). The GSSG/GSH ratio in homozygous Cx46fs380 lenses was not significantly different from wild-type in the cortex at 2.5 months and 4.5 months of age (*p* = 0.07 and *p* = 0.18, respectively), nor did they differ in the nucleus at 2.5 months of age (*p* = 0.45).

We also determined GSSG levels and the GSSG/GSH ratio in samples from lens cortex and nucleus of Cx46fs380 mice. At 2.5 months, there were no significant differences in the GSSG levels or GSSG/GSH ratios regardless of region or genotype (Figure 6A,C). At 4.5 months, the GSSG levels and GSSG/GSH ratio in cortex samples did not differ regardless of genotype (Figure 6B,D). However, the GSSG levels (like GSH levels) were significantly reduced in the nuclei of both heterozygous and homozygous mutant lenses (Figure 6B). The GSSG/GSH ratio significantly increased in the lens nucleus of 4.5-month-old homozygous (but not heterozygous) Cx46fs380 mice (Figure 6D).

### 3.5. Carbonylation of Lens Proteins Is Increased in Cx50D47A, but Not in Cx46fs380 Lenses

**Figure 7:**
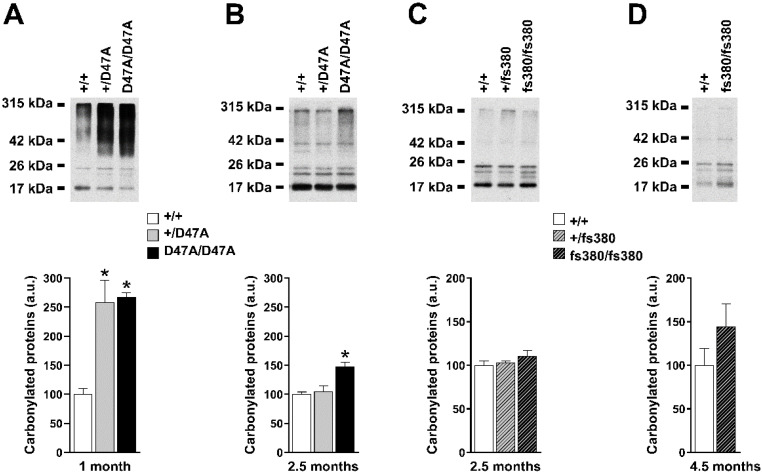
Carbonylated proteins in Cx50D47A and Cx46fs380 lenses. (**A**,**B**). Top panels. Immunoblots of DNP-derivatized proteins in whole lens homogenates from 1- (**A**) and 2.5- (**B**) month-old mice that were wild type for Cx50 (+/+), or heterozygous (+/D47A) or homozygous (D47A/D47A) for Cx50D47A. The migration positions of the molecular mass markers are indicated on the left. Bottom panels. Graphs show the densitometric values of the DNP-derivatized protein bands obtained in three independent experiments. Data are presented as the mean (bar) + standard error of the mean. (**C**,**D**). Top panels. Immunoblots of DNP-derivatized proteins in samples of whole lens homogenates from 2.5- (**C**) and 4.5- (**D**) month-old mice that were wild type for Cx46 (+/+), or heterozygous (+/fs380) or homozygous (fs380/fs380) for Cx46fs380. Bottom panels. Graphs show the densitometric values of the DNP-derivatized protein bands obtained in three independent experiments. Data are presented as the mean (bar) + standard error of the mean. Asterisk denote a significant difference compared with wild-type lenses (*p* < 0.05).

Oxidation can lead to several kinds of protein modifications. We assessed the levels of protein carbonylation in Cx50D47A and Cx46fs380 lenses. Carbonylated proteins in lens homogenates were detected by immunoblotting after DNP-modification of their carbonyl groups. Some carbonylated proteins were detected in all samples independent of genotype. Levels of carbonylated proteins were ~2.5-fold higher in Cx50D47A lenses than in wild-type lenses at 1 month of age (Figure 7A). At 2.5 months, the levels of carbonylated proteins were significantly elevated (by ~50%) only in homozygous Cx50D47A lenses (Figure 7B). The molecular mass range distribution of carbonylated proteins differed between 1- and 2.5-month-old Cx50D47A lenses. A variety of carbonylated proteins with higher molecular mass were more prominent at 1 month than at 2.5 months of age; at 2.5 months, most (but not all) of the carbonylated proteins had lower molecular masses (consistent with crystallins). In Cx46fs380 lenses, the levels and the molecular mass range distribution of carbonylated proteins were similar to wild-type lenses at both 2.5 and 4.5 months of age (Figure 7C,D). 

### 3.6. Cx50D47A and Cx46fs380 Lenses Show Some Increases in High Molecular Weight Proteins

**Figure 8:**
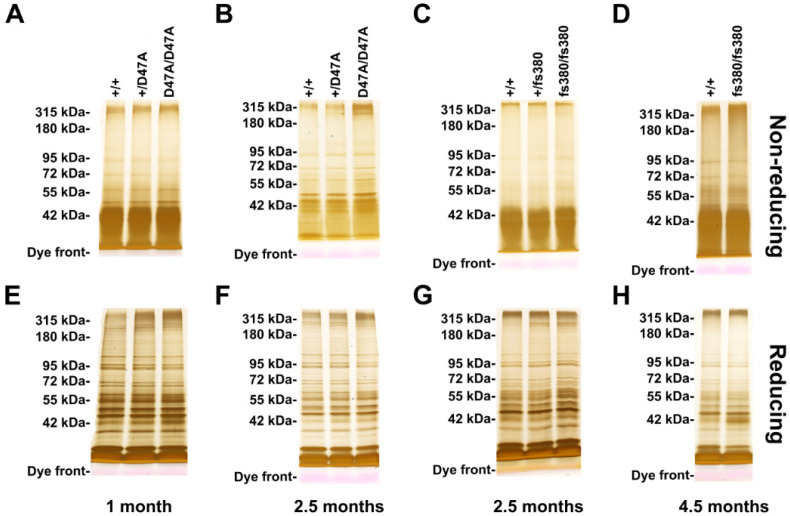
Silver stain detection of proteins in homogenates of Cx50D47A and Cx46fs380 lenses resolved under non-reducing and reducing conditions. (**A**,**B**,**E**,**F**) Images show the pattern of silver stained proteins of whole lens homogenates from 1- (**A**,**E**) and 2.5- (**B**,**F**) month-old mice that were wild type for Cx50 (+/+), or heterozygous (+/D47A) or homozygous (D47A/D47A) for Cx50D47A resolved by SDS-PAGE on 8% polyacrylamide gels under non-reducing (**A**,**B**) and reducing (E,F) conditions. (**C**,**D**,**G**,**H**) Images show the pattern of silver stained proteins of whole lens homogenates from 2.5- (**C**,**G**) and 4.5- (**D**,**H**) month-old mice that were wild type for Cx46 (+/+), or heterozygous (+/fs380) or homozygous (fs380/fs380) for Cx46fs380 resolved by SDS-PAGE on 8% polyacrylamide gels under non-reducing (**C**,**D**) and reducing (**G**,**H**) conditions.

In many cases, cataracts are associated with protein cross-linking and formation of high molecular weight (HMW) aggregates [3,4,5,24]. To look for such changes in the connexin mutant lenses, we subjected homogenates of wild type and heterozygous or homozygous Cx50D47A and Cx46fs380 lenses to SDS-PAGE under non-reducing and reducing conditions, and used silver staining to detect the proteins. In all samples, the banding patterns looked relatively similar; however, there were some apparent differences in the intensity of the silver staining in the regions containing the high molecular weight (HMW) proteins (Figure 8). We quantified the bands in these regions by determining the mean grey value within a box encompassing the proteins that migrated with the slowest mobilities (M_r_ > 180 kDa) (Table 2). Under non-reducing conditions, the abundances of HMW proteins in 1- and 2.5-month-old Cx50D47A lenses were similar to wild type (Figure 8A,B and Table 2). The apparent increase in abundance of non-reduced HMW proteins in 2.5-month-old homozygous Cx50D47A lenses did not reach statistical significance (*p* = 0.058). In contrast, under reducing conditions HMW proteins were significantly increased in 1-month-old homozygous Cx50D47A lenses (by 42% vs. wild type) and in both heterozygous and homozygous Cx50D47A lenses at 2.5 months of age (by 23% and 50%, respectively). There were no differences in abundance of HMW proteins between 2.5-month-old wild type and Cx46fs380 lenses under either non-reducing or reducing conditions (Figure 8C,G and Table 2), although under reducing conditions a protein band of ~250 kDa seemed more prominent in Cx46fs380 mutant lenses than in wild type lenses (Figure 8G). At 4.5 months of age, HMW proteins were significantly increased in Cx46fs380 homozygous lenses (by 14%) under non-reducing conditions, but showed no significant differences under reducing conditions (Figure 8D,H, and Table 2).

## 4. Discussion

In this study, we have reported the levels of reduced and oxidized GSH and oxidation status of proteins (carbonylation and presence of β-mercaptoethanol-reducible bonds in HMW proteins) in Cx50D47A and Cx46fs380 lenses. Interestingly, the time course and direction of the alterations in GSH levels and the oxidative changes in lens proteins differed between the two mouse lines. Some of our observations are rather surprising, because they contradict the expected results based on previous publications suggesting a general causative relationship between decreased GSH, increased oxidation, and cataract development. 

The concentration of GSH is very high in the lens [5,7,25], favoring the maintenance of protein thiol groups in their reduced state. The GSH levels that we measured in wild-type mouse lenses are consistent with previously reported values [22]. Like other investigators, we observed that GSH levels are much higher in the cortex than in the nucleus of the lens [10,21,22,23]. This difference may result from the previously described GSH diffusion barrier [5,26]. The data presented here suggest that the barrier is independent of gap junctional intercellular communication.

Cataract formation has previously been associated with oxidative stress, declining GSH concentrations, and the formation of protein–thiol mixed disulfides [4,6,8]. However, we did not observe a decrease in GSH levels in whole lens homogenates of any of the mutant mice, including pre-cataractous, transparent lenses (2.5-month-old Cx46fs380 heterozygotes) or lenses containing cataracts (2.5- and 4.5 month-old Cx46fs380 homozygotes and Cx50D47A mice at either of the ages studied). A decrease in GSH was only found in the nuclear region of 4.5-month-old homozygous Cx46fs380 lenses (which have a substantial cataract). Against all expectations, GSH levels were increased in homozygous Cx50D47A lenses at 2.5 months of age (even though these mice have severe cataracts before 1 month of age). Therefore, there is no correlation between reductions of GSH and the presence of cataracts in the connexin mutant mice, implying that they are not causally related. GSH reductions in a lens region did occur in some instances (nuclei of older Cx46fs380 mice) in association with cataract progression/exacerbation. 

Intracellular levels of GSH in the lens are governed in part by the activities of glutathione synthetase and glutathione reductase (which regenerates reduced GSH from GSSG). They are also influenced by a GSH transporter (which facilitates uptake of GSH into cortical fibers) [23,27]. The increase in GSH levels in homozygous Cx50D47A lenses may result from the increased levels of glutathione synthetase and glutathione reductase. Because differentiation is impaired in Cx50D47A lenses [13], this increase in enzyme levels may represent a compensatory response to supply GSH to the remaining organelles. The increased levels of GSH-generating enzymes in the Cx50D47A lenses might also result from the activation of a p62-dependent antioxidant response. Cx50D47A lenses have increased levels and phosphorylation of p62/sequestosome 1 at serine349 [28]. This post-translational modification has been implicated in the antioxidant response pathway by allowing translocation of Nrf2 to the nucleus and stimulation of the expression of protective antioxidant genes [29]. Similarly, the increase in glutathione synthetase levels in homozygous Cx46fs380 lenses at 4.5 months of age may represent an initial step of an antioxidant response to minimize cataract progression. Interestingly, the sequence of steps in the antioxidant response appears to differ between the mouse lines. The increase in levels of glutathione reductase in homozygous Cx50D47A lenses occurred before an increase in glutathione synthetase levels could be detected, whereas no change in glutathione reductase was detectable in Cx46fs380 lenses even at 4.5 months of age. 

Studies of lenses from mice with targeted deletion of Cx46 or Cx50 suggested that the presence of Cx46 (and not of Cx50) is necessary for the transport of GSH from the lens cortical cells (where the enzymes responsible for keeping GSH in high concentration are located) to the lens nuclear cells, even though both Cx46 and Cx50 gap junctions allow permeation of GSH [10]. Decreased influx of GSH through gap junction channels may have contributed to the reduced GSH (and GSSG) levels in the lens nuclei of 4.5-month-old Cx46fs380 mice. However, if all transport of GSH to the lens nucleus depended on Cx46, a decrease in the nuclear concentration of GSH should have been observed much earlier than 4.5 months of age in Cx46fs380 lenses, because Cx46 levels are already extremely low by 1 month of age [12]. However, this was not the case, suggesting that GSH levels in the nucleus of young Cx46fs380 lenses were maintained through a mechanism other than gap junctional intercellular communication. 

Many studies have suggested that oxidative damage to lens proteins and lipids is a major factor in cataract development and that maintenance of the transparent lens is facilitated by high levels of reduced GSH. While a decrease in GSH levels or an increase in GSSG levels can be indicative of an oxidizing environment, the GSSG/GSH ratio gives a more specific indication of alterations in redox metabolism. The increases in the GSSG/GSH ratio in the nuclear region of 4.5-month-old homozygous Cx46fs380 lenses and in whole Cx50D47A lenses indicate that the lenses of both mouse lines have a more oxidizing environment than wild-type lenses. However, the alteration in redox metabolism (as evidenced by the increase in the GSSG/GSH ratio) was much greater in Cx50D47A whole lenses than in Cx46fs380. 

Proteins in the lens are subject to changes and modifications, including oxidation [24,30]. Oxidation of lens components has been widely studied [7,8,31]. The change in redox metabolism in the nucleus of homozygous Cx46fs380 lenses did not lead to detectable changes in the pattern or levels of lens carbonylated proteins, but it may be responsible for the small increase of HMW proteins containing β-mercaptoethanol-reducible bonds (likely protein–protein or protein–thiol mixed disulfide bonds). Glutathionylation of proteins may also have contributed to the decrease in GSH levels in the lens nucleus of 4.5-month-old Cx46fs380 and to the formation of HMW proteins in these lenses. In contrast, the strong oxidizing environment in Cx50D47A lenses is likely responsible for their significant increase in protein carbonylation. Indeed, the temporal pattern of the GSSG/GSH ratio in these lenses parallels that of protein carbonylation (being more severe at 1 month than at 2.5 months). Surprisingly, the carbonylation of proteins in Cx50D47A lenses appeared to decrease (instead of increase) with age (see Figure 7). Because the lens continues to grow and the concentration of non-modified low molecular weight crystallins increases during cell maturation, the decrease in levels of carbonylated proteins may result in part from a dilution effect. Alternatively, the decrease could be due to decarbonylation, since it has recently been demonstrated that protein carbonylation is not irreversible [32,33]. However, considering the oxidizing environment of Cx50D47A lenses, it is likely that the reduction in levels of carbonylated proteins results from their degradation, since this modification can target proteins for degradation [34,35,36].

Our studies detecting and quantifying HMW proteins under non-reducing and reducing conditions were designed to assess changes in covalent cross-linking of proteins in the mutant mice. Under non-reducing conditions, we only detected an increase in HMW proteins in homozygous Cx46fs380 lenses at 4.5 months of age (but not earlier), and did not find differences in HMW protein content in Cx50D47A lenses at either age studied. The absence of detectable changes in disulfide linked proteins, except in the older Cx46fs380 lenses, adds to the evidence that oxidation of sulfhydryl groups has a limited role in cataractogenesis in these animals. In contrast, under reducing conditions, Cx50D47A lenses showed increases in HMW proteins at both 1 month (homozygotes) and 2.5 months (heterozygotes and homozygotes) suggesting an increase in a different form of covalent protein crosslinking. 

## 5. Conclusions

In summary, our data suggest that connexin mutant cataracts do not result from loss of intercellular passage of GSH. Although lenses of both Cx46fs380 and Cx50D47A mice contain some oxidative damage as seen in other kinds of cataracts, our data suggest that oxidation is not a critical early factor for cataract development (at least in the Cx46fs380 lenses). It is likely that other factors, such as impaired intercellular circulation of calcium ions [15,16] and cellular stress [19], play greater roles.

## Figures and Tables

**Table 1:** Differences between the characteristics of Cx46fs380 and Cx50D47A lenses *.

	Cx46fs380	Cx50D47A
**Age of Noticeable Cataract**	~2 months homozygotes; ≥4 months heterozygotes	<1 month heterozygotes and homozygotes
**Differentiation**	Normal	Impaired
**Lens Size**	Normal	Smaller than wild-type
**Severity of Cataract**	Less severe compared with Cx50D47A cataracts	Severe

* Based on data published in [12,13].

**Table 2:** High molecular weight protein content under non-reducing and reducing conditions.

Age	Genotype	Mean Grey Value (Mean ± S.E.M.)		Number of Sets (*n*)
		Non-reducing	Reducing	
1 month	+/+	52.1 ± 6.4	57.9 ± 10.0	4
	+/D47A	52.5 ± 7.0	68.4 ± 10.3	
	D47A/D47A	60.0 ± 7.1	82.3 ± 10.3 *	
2.5 months	+/+	35.1 ± 3.3	47.5 ± 1.3	3
	+/D47A	37.3 ± 4.2	58.6 ± 0.6 *	
	D47A/D47A	64.3 ± 8.3	71.2 ± 2.1 *	
2.5 months	+/+	41.1 ± 5.2	64.8 ± 7.0	3
	+/fs380	38.0 ± 4.0	79.4 ± 3.2	
	fs380/fs380	42.1 ± 1.9	76.4 ± 2.2	
4.5 months	+/+	92.5 ± 1.7	78.6 ± 5.6	3
	fs380/fs380	105.3 ± 3.4 *	77.9 ± 4.0	

* Denotes a significant difference compared with wild-type lenses (*p* < 0.05).
